# Total Hip Replacement in Severe Haemophilia A: Challenges and Feasibility

**DOI:** 10.7759/cureus.29847

**Published:** 2022-10-02

**Authors:** Sujoy K Bhattacharjee, Farhan Naim

**Affiliations:** 1 Orthopedics, Sarvodaya Hospital and Research Centre, Faridabad, IND; 2 Hematology, Sarvodaya Cancer Institute, Faridabad, IND

**Keywords:** hemarthrosis, total joint arthroplasty, male, adults, haemophilia a

## Abstract

The management of haemophilic patients is complicated due to multiple comorbidities. A dedicated haemophilia treatment centre with a multidisciplinary team can plan and execute elective orthopaedic surgery in such haemophilic individuals.

A cementless total hip arthroplasty (THA) was performed in a 26-year-old male patient with haemophilic arthropathy of the right hip under factor VIII replacement therapy based on activated partial thromboplastin time levels. The patient received a preoperative dose of recombinant anti-haemophilic factor. Venous thromboembolic event prophylaxis was not given. Postoperative radiographs demonstrated successful prosthesis placement. The patient could bear weight and walk unassisted two weeks after surgery.

THA in patients with haemophilia leads to significant improvement in joint function with a relatively low incidence of complications with modern techniques and haematological management.

## Introduction

Haemophilia A is an X-linked recessive disease due to a deficiency of clotting factor VIII. Haemophilia A is quite prevalent with approximately one in 5000 live male births [1].

The joints most frequently affected by haemophilic arthropathy (HA) are the knee, elbow, and ankle [2]. The severity of haemophilia can range from severe (factor levels less than 1%) to moderate (factor levels of 1-5%) to mild (factor levels of 6-30%) based on the deficiency of coagulation factor VIII in the blood. It is expressed in percentage (100% represents the normal concentration for the investigated coagulation factor).

The management of haemophilic patients is complicated due to multiple comorbidities. Joint problems developing due to recurrent haemarthrosis, such as chronic synovitis and degenerative arthritis, serve as a significant cause of morbidity. The incidence of haemarthrosis increases with age, with 21% of haemophiliac children reportedly belonging to age one to six years, which increases to 50% in those aged 10-17 years, followed by up to 60% in those aged 18-65 years [3-5].

A dedicated haemophilia treatment centre with a multidisciplinary team can plan and execute elective orthopaedic surgery in such haemophilic individuals. The first reported hip arthroplasty in a haemophiliac patient was done in 1967 [6]. However, the fixation methods of components (i.e., cemented or uncemented) have been controversial for haemophilic hips [7]. The team should include a haematologist, an expert arthroplasty surgeon, a rehabilitation physician, and a physiotherapist. The most prevalent issues are postoperative bleeding, poor bone quality, and muscular contracture [8,9]. Here, we report a cementless total hip arthroplasty (THA) performed in a 26-year-old patient with HA of the right hip under factor VIII replacement therapy based on activated partial thromboplastin time (APTT) levels. Written informed consent was obtained from the patient after informing the purpose in his local language.

The surgical challenges arising in THA in haemophilia are abnormal bleeding, concurrent viral disease, difficulty in achieving successful fixation at the bone and implant interface, abnormal femoral and acetabular anatomy soft tissue contractures, and poor bone quality [10].

## Case presentation

A 26-year-old male patient experiencing recurrent haemarthrosis for the past one year, involving the knee and elbow joints, presented with severe pain and stiffness of the right hip joint. Complete blood count (CBC), prothrombin time (PT), APTT, and clotting factor tests were done to determine the clotting factors level (Table 1).

**Table 1:** Baseline parameters

Parameter	Value
Haemoglobin	12.0 gm/dl
Total leucocyte count (TLC)	5680/mm2
Platelet count	1.56 lakhs/cum
Activated partial thromboplastin time	102 s (Mn 36 s)
Prothrombin time	12.5 s (Mn 12 s)
Factor eight levels	0.5%
Bethesda assay	No inhibitors

All these tests were initially done by a haematologist, and based on the normal values obtained in these tests, the haematologist gave us a yes for the surgery. The patient was diagnosed at an early stage by a haematologist for haemophilia A. Parameters were measured throughout the surgery by the haematologist and consulting physician. All the necessary tests were conducted to rule out any associated medical conditions. On further examination, his right hip was found ankylosed with global restriction of range of motion. The patient could bear weight only with support and was non-ambulatory for three months before surgery.

**Figure 1:**
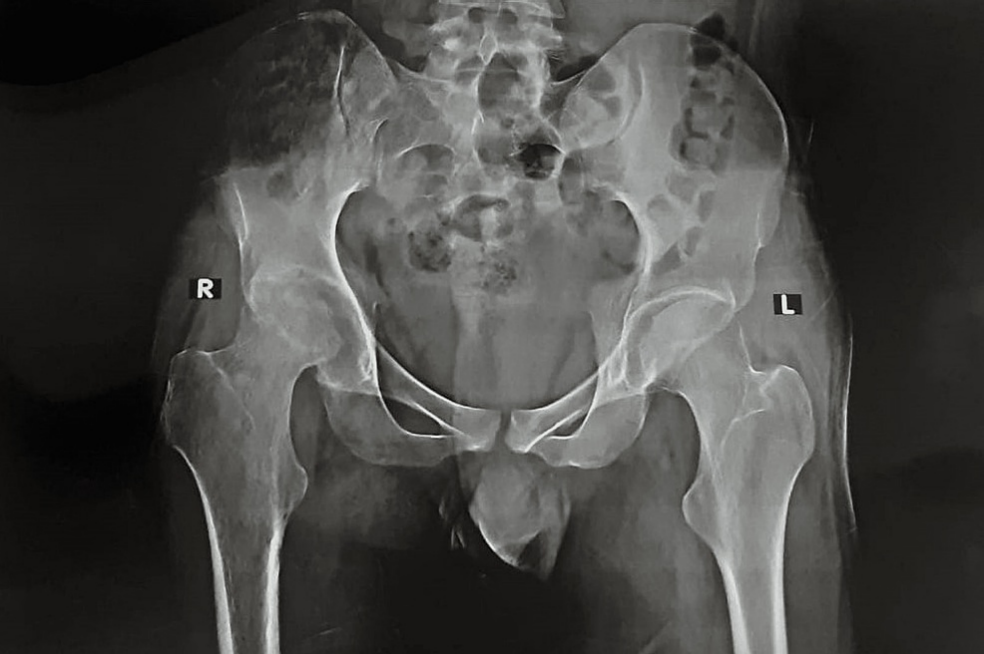
Preoperative X-ray

Radiographic examination showed end-stage destruction of the right hip joint. Arnold and Hilgartner’s [2] classifications and Pettersson and Ahlberg's [11] scoring system assessed the preoperative radiographic appearance (Table 2 and Figure 1). The score for the right hip analysed by the radiographic scoring system was calculated to be 7.

**Table 2:** Pettersson scoring system

Radiographic parameter	Finding	Score
Osteoporosis	Absent	0
	Present	1
Enlarged epiphysis	Absent	0
	Present	1
Irregular subchondral surface	Absent	0
	Slight	1
	Pronounced	2
Narrowing of joint space	Absent	0
	<50%	1
	>50%	2
Subchondral cyst formation	Absent	0
	One cyst	1
	More than one cyst	2
Erosions at joint margins	Absent	0
	Present	1
Gross incongruence of articulating bone ends (angulation/displacement)	Absent, slight, pronounced	0, 1, 2
	Slight	1
	Pronounced	2
Joint deformity	Absent	0
	Slight	1
	Pronounced	2

He had no prior history of being treated with a factor VIII inhibitor, and his baseline factor VIII level was 0.5%. Hepatitis C virus (HCV) and HIV were negative. To relieve the joint dysfunction and pain in the hip joint, total hip replacement (THR) was planned. Standardised preoperative, intraoperative, and postoperative protocols were required and needed to be adjusted due to severe haemophilia A and the associated high risk of bleeding and infection. As a result, a team-based approach was used to optimise the patient's clinical management. An inhibitor screen using a standard Bethesda assay showed the absence of inhibitors.

Haemophilia management

The patient received a preoperative dose of recombinant anti-haemophilic factor (120% correction) before surgery. The risk of bleeding was greater than the risk of either a deep venous thrombosis (DVT) or a fatal pulmonary embolism, so pharmacologic venous thromboembolism (VTE) prophylaxis was not given. However, intermittent pneumatic compression devices (IPCDs) were used both during and after surgery. For excessive bleeding, an additional dose of 20% recombinant factor VIII was administered intraoperatively.

Recombinant factor VIII infusions were reduced to once every 12 hours on postoperative day one and titrated according to activated PT. After three days of postoperative 60% correction and three days of 40% correction, the dose was reduced to once daily with 80% correction until patient hospitalisation (D + 10). Haemoglobin stabilisation, postoperative haemostasis, and a normal activated PT predicted discharge.

The patient was operated in June 2021 during the second coronavirus disease 2019 (COVID-19) wave. Due to the travel restriction in India, it was not possible for him to commute from his remote hometown to get a radiological examination for possible follow-ups.

Operative details

**Figure 2:**
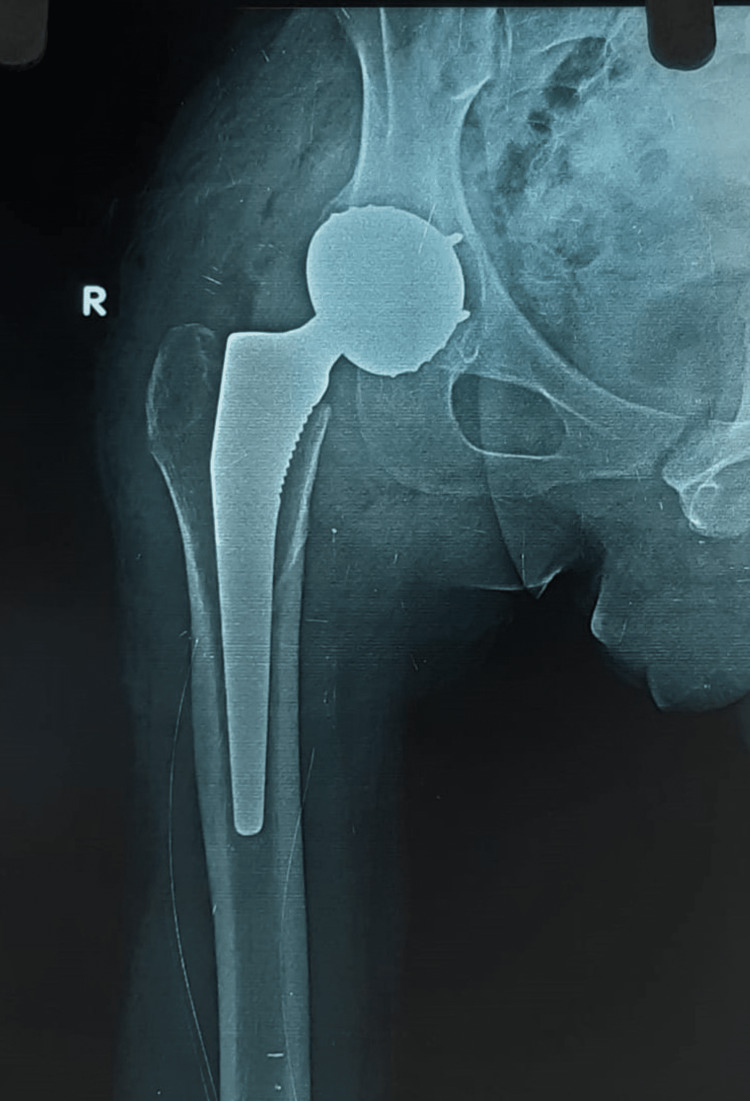
Immediate postoperative X-ray

Before surgery, the patient received 1.5 grams of cefoperazone sulbactam, 600 mg linezolid, and 1 gram of tranexamic acid intravenously. The procedure was done in the presence of a haematologist. Moore's technique was used in a left lateral position. Intraoperatively, one unit of packed red blood cells (PRBC) was transfused. Uncemented acetabular and femoral implants were used. The cup was made of stainless steel (SS) and coated with hydroxyapatite. The stem was titanium and coated with hydroxyapatite. The bearing couple was 28 mm SS inserted in the poly-dual mobility construct, and all the implants were of evolution. Extensive soft tissue release was done on the femoral side; no adductor tenotomy was done. Gluteus maximus insertion release was done. Short external rotators were not repaired because they were very fragile and had lost their strength. Tranexamic acid injection (5 ml) and 2 grams of vancomycin powder were poured inside the joint. The closure was done in layers, and skin closure was done with barbed Monocryl sutures. After the surgery, IPCDs were appropriately applied. Postoperative radiographs demonstrated successful prosthesis placement (Figure 2).

Assisted gait training was started 24 hours after surgery with full weight-bearing, and guarded muscle strengthening exercises were also initiated on the day after surgery followed by physiotherapy. The patient could bear weight and walk unassisted two weeks after surgery. After 16 weeks, the patient was even able to drive a car.

**Figure 3:**
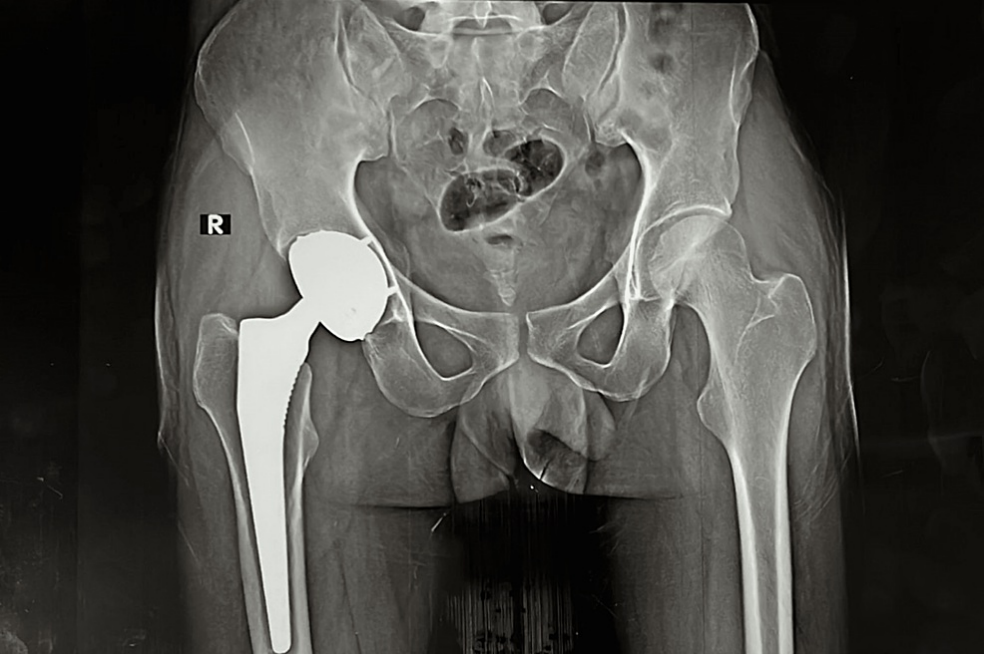
Sixteen weeks postoperative X-ray

During the routine clinical and radiological evaluation, no signs of implant loosening, infection, or other complications of the implant components were observed (Figure 3).

## Discussion

HA is characterized by intra-articular haemorrhage rather than systemic inflammation as in rheumatoid arthritis or age-related deterioration as in osteoarthritis [9]. The pathobiological mechanisms that cause hypertrophic synovium, hypervascularity, and cartilage destruction are still unknown [12].

THA is a successful treatment for end-stage HA of the hip with proper prophylaxis and clotting factor replacement. Severe, debilitating pain with exercise and restricted joint function is a THA indication in hip HA patients [7]. Past studies have shown that cemented THA failure rates have been higher than uncemented ones, attributed to the early loosening through microhaemorrhages at the bone-cement interface caused by haemorrhagic diathesis [7].

Strauss et al. studied 45 haemophilic individuals with THA [13] and found that the mean Harris hip score increased from 38.5 to 82.1 postoperatively. In our situation, the patient's hip score went from 16 preoperative (bedridden for eight months) to 81 at four months postoperatively (was able to drive).

In our study, the clotting factor consumption was 12,000 U, significantly less than the study conducted by Wu et al. in 2017 [14]. The mean amount of clotting factor VIII used in the perioperative period to manage haemophilia A was 14,031.3 U.

The typical blood loss is 300-500 ml in patients undergoing THA for osteoarthritis without clotting abnormalities [15,16]. In our case, the blood loss was 450 ml, whereas Wu et al. [14] reported a blood loss ranging from 1494 to 7506 mL in 24 haemophilic patients who underwent THA. A retrospective study by Colgan et al. (10-year duration) on 12 haemophilia patients who underwent THA reported a mean blood loss of 502 ml [17].

While DVT prevention is typical in most THA patients, the danger of bleeding must be evaluated against the risk of VTE in the haemophilia population. Extensive bleeding occurs in roughly 50% of haemophilic individuals undergoing THA or total knee arthroplasty with low-molecular-weight heparin. Regardless of prophylaxis, 39.1% of haemophilic patients undergoing total joint arthroplasty experience significant bleeding [18]. IPCDs and early ambulation are two other non-pharmacologic DVT prevention methods. It is challenging to balance surgical site bleeding versus DVT/pulmonary embolism. We used IPCDs and early ambulation because the risk of major bleeding and infection outweighed the risk of VTE.

Based on the reviews obtained from studies done so far, newer cementless implants have higher bioactivity in osteointegration than older components. Recent research demonstrates that cementless THA outperforms cemented THA in haemophilia patients with fewer problems and longer prosthesis life [19]. Given the patient's age and the good results of the new generation cementless THAs, we chose a cementless metal-on-polyethylene coupling in this case.

## Conclusions

One should medically evaluate the patient’s well-being and take great care to avoid the complications of major bleeding or infection by optimizing the levels of factor VIII in the perioperative period. During surgery, accurate haemostasis must be obtained at every step of the surgical procedure with the help of electrocautery and tranexamic acid. One should likely avoid VTE chemoprophylaxis in low-risk patients. A cementless THA in a young patient with severe haemophilic hip arthropathy can achieve a satisfactory result without complications in a short follow-up period. A successful arthroplasty and adequate replacement of clotting factors combined with a meticulous team approach are the key factors in ensuring good outcomes.

THA in haemophilia patients with modern techniques and haematological management results in significant improvement in joint function with a relatively low incidence of complications. Increased blood loss and substitution therapy have no obvious negative influence on the mid-term to long-term results of THA.

## References

[1] Berntorp E, Shapiro AD (2012). Modern haemophilia care. Lancet.

[2] Arnold WD, Hilgartner MW (1977). Hemophilic arthropathy. Current concepts of pathogenesis and management. J Bone Joint Surg Am.

[3] Carulli C, Felici I, Martini C, Civinini R, Linari S, Castaman G, Innocenti M (2015). Total hip arthroplasty in haemophilic patients with modern cementless implants. J Arthroplasty.

[4] Zhai J, Weng X, Lin J, Qian W, Guo S (2017). Efficacy of a modified coagulation factor substitution for total hip arthroplasty in patients with end-stage haemophilic arthropathy. Blood Coagul Fibrinolysis.

[5] Mann HA, Choudhury MZ, Allen DJ, Lee CA, Goddard NJ (2009). Current approaches in haemophilic arthropathy of the hip. Haemophilia.

[6] Habermann B, Eberhardt C, Hovy L, Zichner L, Scharrer I, Kurth AA (2007). Total hip replacement in patients with severe bleeding disorders. A 30 years single center experience. Int Orthop.

[7] Kelley SS, Lachiewicz PF, Gilbert MS, Bolander ME, Jankiewicz JJ (1995). Hip arthroplasty in hemophilic arthropathy. J Bone Joint Surg Am.

[8] Mortazavi SJ, Bagheri N, Farhoud A, Hadi Kalantar S, Ghadimi E (2020). Total knee arthroplasty in patients with hemophilia: what do we know?. Arch Bone Jt Surg.

[9] Melchiorre D, Manetti M, Matucci-Cerinic M (2017). Pathophysiology of hemophilic arthropathy. J Clin Med.

[10] Miles J, Rodríguez-Merchán EC, Goddard NJ (2008). The impact of haemophilia on the success of total hip arthroplasty. Haemophilia.

[11] Pettersson H, Ahlberg A, Nilsson IM (1980). A radiologic classification of hemophilic arthropathy. Clin Orthop Relat Res.

[12] Wyseure T, Mosnier LO, von Drygalski A (2016). Advances and challenges in hemophilic arthropathy. Semin Hematol.

[13] Strauss AC, Rommelspacher Y, Nouri B (2017). Long-term outcome of total hip arthroplasty in patients with haemophilia. Haemophilia.

[14] Wu GL, Zhai JL, Feng B, Bian YY, Xu C, Weng XS (2017). Total hip arthroplasty in hemophilia patients: a mid-term to long-term follow-up. Orthop Surg.

[15] Bromhead H (2007). Anaesthesia for hip arthroplasty. ATOTW.

[16] Austin MS, Hozack WJ, Sharkey PF, Rothman RH (2003). Stability and leg length equality in total hip arthroplasty. J Arthroplasty.

[17] Colgan G, Baker JF, Donlon N, Hogan N, McCarthy T (2016). Total hip arthroplasty in patients with haemophilia - what are the risks of bleeding in the immediate peri-operative period?. J Orthop.

[18] Pathak N, Munger AM, Charifa A, Laskin WB, Bisson E, Kupfer GM, Rubin LE (2020). Total knee arthroplasty in hemophilia A. Arthroplast Today.

[19] Maggs J, Wilson M (2017). The relative merits of cemented and uncemented prostheses in total hip arthroplasty. Indian J Orthop.

